# Susceptibility artifacts induced by crowns of different materials with prepared teeth and titanium implants in magnetic resonance imaging

**DOI:** 10.1038/s41598-021-03962-w

**Published:** 2022-01-10

**Authors:** Xiaomeng Gao, Qianbing Wan, Qingping Gao

**Affiliations:** 1 Department of Stomatology, Xiangya Hospital, Central South University, 87 Xiangya Road, Changsha, 410008 Hunan China; 2 State Key Laboratory of Oral Diseases, National Clinical Research Center for Oral Diseases, Department of Prosthodontics, West China Hospital of Stomatology, Sichuan University, Chengdu, 610041 China

**Keywords:** Dentistry, Medical imaging

## Abstract

This study aimed to investigate the artifacts induced by crowns composed of different materials with prepared teeth and titanium implants. Resin, metal-ceramic, ceramic and zirconia crowns were fabricated and placed onto the prepared teeth on a human cadaver head or titanium implants with prosthesis abutments on a dry human mandible. The samples were scanned on a 1.5 T MRI apparatus, and artifact areas were defined as the signal intensity and signal loss adjacent to the prosthesis and measured by a threshold tool with ImageJ2x. Data were analyzed using SPSS 22.0. Resin, ceramic, zirconia, and precious metal-ceramic crowns barely produced artifacts on the cadaver skull (*p* > 0.999). By contrast, pure Ti and nonprecious metal-ceramic crowns created significant artifacts (*p* < 0.001). The average artifacts reduction of double Au-Pt and Ag-Pd metal-ceramic crowns combined with titanium implants and abutments was 79.49 mm^2^ (*p* < 0.001) and 74.17 mm^2^ (*p* < 0.001) respectively, while artifact areas were increased in double Co-Cr and Ni–Cr metal-ceramic crowns by 150.10 mm^2^ (*p* < 0.001) and 175.50 mm^2^ (*p* < 0.001) respectively. Zirconia, ceramic and precious metal-ceramic crowns induce less MRI artifacts after tooth preparation while precious metal-ceramic crowns alleviate artifacts in combination with titanium implants.

Magnetic resonance imaging (MRI) of the head and neck is widely used to diagnose tumours and other lesions in the oral cavity, temporomandibular joint and salivary gland^1–5^. However, susceptibility artifacts, also referred to as metal artifacts, are caused by differences in susceptibility between a prosthesis and the adjacent tissue, thereby inducing inhomogeneity in a magnetic field^6^. Metallic prostheses, such as different kinds of full crowns and titanium implant restorations may distort images during MRI examinations and hence interfere with clinical diagnosis^7–9^.

Crowns, bridges and implant restorations are commonly-used fixed prosthesis and have been the major treatment options in prosthodontics. New inventions and innovations have brought new materials with improved physiochemical and aesthetic performances to satisfy patients’ needs^10^. All-ceramic crowns have become a superior treatment alteration to metal-ceramic crowns with better aesthetic properties and comparable survival rates^11,12^. Titanium implants are also stable and well-known prostheses for missing teeth and have gained clinical preference because of their desirable biomechanics, biocompatibility and non-invasive properties^13,14^.

Some studies have found that dental materials could cause different metal artifacts in MRI mainly depending on their susceptibilities^15–17^. A recent study has investigated the artifacts of three kinds of commercial dental spherical alloys with different diameters in a Ni-doped agarose solution^18^. This study revealed that the susceptibility artifacts of alloys decreased with the magnetic susceptibility. However, the investigations on artifacts caused by all-ceramic crowns are insufficient. Besides, the shape of the materials also influenced the susceptibility artifacts in MRI^19^. Most of the studies have applied the spherical or cylindrical materials and only a few have taken the shape of the crowns into consideration^18,20,21^. Studies have been conducted to investigate the MR artifacts of titanium implants^9,15,22^. Titanium implants were generally accepted to induce mild to moderate artifacts compared with other metallic prostheses^7,20^. However, the implant restorations are combined with prosthesis abutments, screws and crowns in clinical setting. A study has investigated the artifacts induced by crowns of different materials with titanium implants and revealed that precious alloy- and zirconia-based implant-supported crowns could induced the least MRI artifacts^23^. Besides, a research has proposed that combining two materials of different magnetic susceptibility (diamagnetic and paramagnetic) has the desired effective magnetic susceptibility artifacts^6^. Based on their results, we included more kinds of crowns of different materials and tried to clarify the reason for the susceptibility artifacts caused by the different materials^23,24^.

As such, this research initially investigated the areas and distribution patterns of susceptibility artifacts induced by commonly-used dental crowns in MRI. We hypothesized that ceramic, zirconia and precious metal-ceramic crowns would produce insignificant artifacts on the prepared teeth, while nonprecious metal-ceramic crowns would induce significant artifacts in MRI compared with resin crowns. In addition, when combined with titanium implants, artifact size would be reduced with diamagentic metal-ceramic crowns and increased with paramagnetic metal-ceramic crowns. Besides, this work aimed to compare and analyze the artifacts of crowns alone and those combined with implants in a simulated anatomical environment. Understanding of the areas and distribution patterns of susceptibility artifacts would lead to a better knowledge of the influence on diagnosis and help to a comprehensive selection of dental materials.

## Methods

### Crown and implant materials

Nine kinds of crown materials and two titanium implants with prosthesis abutments were selected to cover a broad spectrum of clinically used prostheses. The detailed information on these materials is presented in Table 1.

**Table 1:** Composition and manufacturers of used crowns and implants.

Samples	Main composition	Vendor
Resin crown	Acrylic resin, polymethyl methacrylate	Shanghai Second Hospital (Shanghai, China)
Ni–Cr metal-ceramic crown	Nickel: 58%–60%, chromium: 22%–24%, molybdenum: 9%–10%, niobium: 1.5%–2.5%, silicon: 1.5%–2.5%	Harbin Qianluda Medical instrument Co., Ltd. (Harbin, China)
Ag-Pd metal-ceramic crown	Silver: 46%, palladium: 42.9%, gold: 2%, indium: 8%	Grikin Advanced Material Co., Ltd. (Beijing, China)
Pure Ti metal-ceramic crown	Titanium: 99.64%, oxygen: 0.051%, iron: 0.016%	Shanxi Xijing Medical instrument Co., Ltd. (Shanxi, China)
Co-Cr metal-ceramic crown	Cobalt: 61%, chromium: 26%, molybdenum: 5%, tungsten: 5%, silicon: 1%	American Argen Corporation (San Diego, USA)
Au-Pt metal-ceramic crown	Gold: 82.6%; platinum: 11.5%; zinc: 1.5%; tantalum, ruthenium, manganese ≤ 0.1%	Grikin Advanced Material Co., Ltd. (Beijing, China)
Ti alloy metal-ceramic crown	Nickel: 58%, chromium: 24%, molybdenum: 10%, titanium: 4%–6%, silicon: 1%	Shanxi Xijing Medical Instrument Co., Ltd. (Shanxi, China)
Zirconia crown	Zirconia: 94%–95%, iridium oxide: 4.5%-5.5%, alumina ≤ 0.5%	Liaoning Aierchuang Biomedical Material Co., Ltd. (Liaoning, China)
Ceramic crown	Silica: 70%, potassium oxide: 12%, lithium oxide: 11%, magnesium oxide (5%), other oxides (2%)	Harbin Qianluda Medical Instrument Co., Ltd. (Harbin, China)
Titanium implants (length: 8 mm, diameter: 4.1 mm)/prosthesis abutments	Titanium based on ASTMF67, oxygen: < 0.4%, iron: < 0.2%, carbon: < 0.08%, nitrogen: < 0.03%, hydrogen: < 0.015%	Straumann (Basel, Switzerland)

For all metallic porcelain crowns, the components above only include the metallic parts, and their porcelain parts were composed of silicon dioxide [52%–62%], alumina [11.6%–13.5%], potassium oxide [9.6%–11.3%] and sodium oxide [4.75%–5.37%].

### Phantom design

The study was conducted following the guidelines of Helsinki Declaration and with permission from the Medical Ethic Committee of Xiangya Hospital of Central South University (approval number: 201610068).

**Figure 1:**
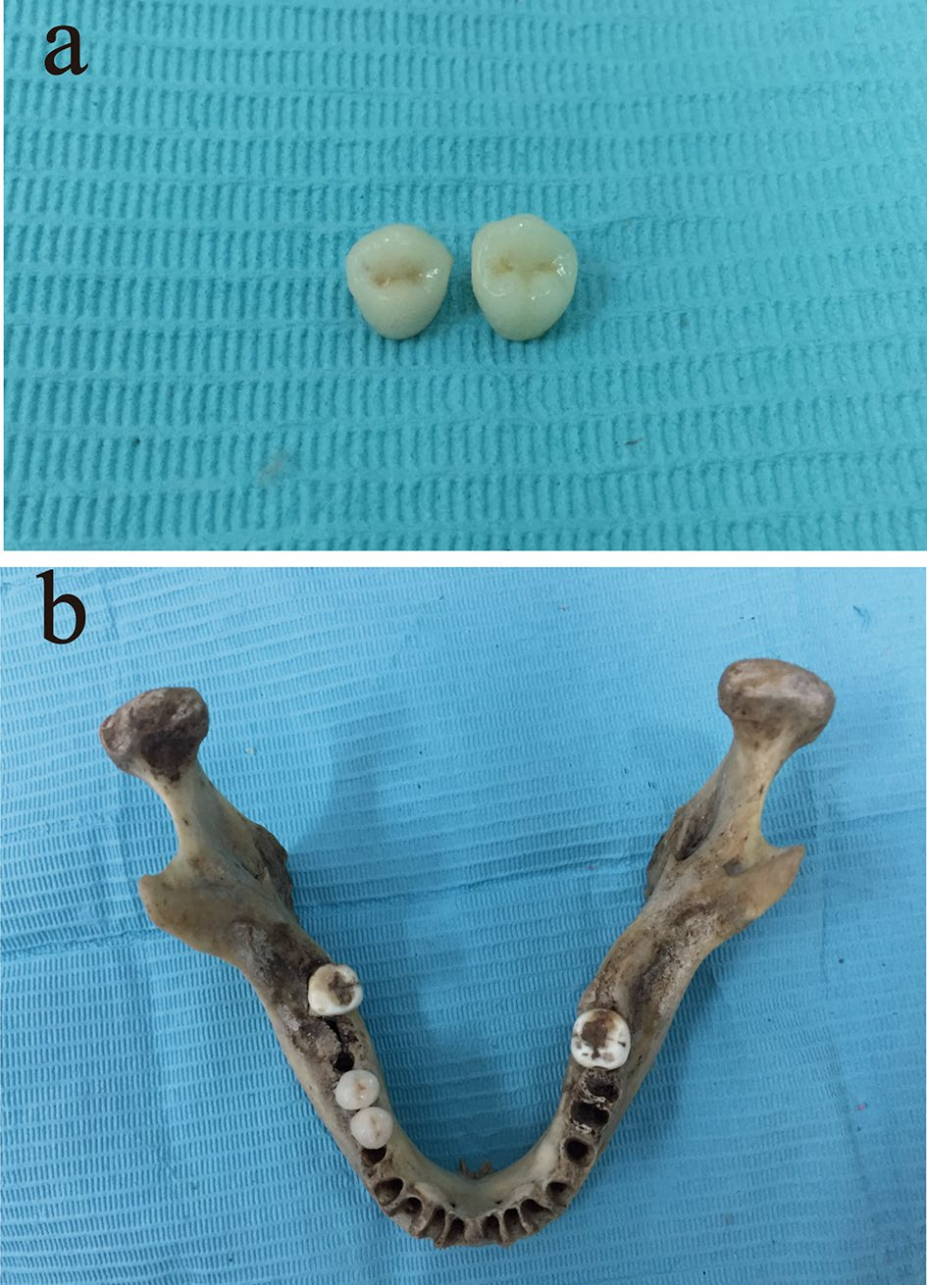
Zirconia crowns of premolars after fabrication (**a**) and a dry human mandible after implant insertion and zirconia crown restoration (**b**).

The cadaver used was collected from Anatomy Laboratory in Central South University with informed consent waiver statement and was approved for use in the study from the Medical Ethic Committee of Xiangya Hospital of Central South University. The dry human mandible was also collected from the Anatomy Laboratory in Central South University. Tooth preparations, silicon rubber (Aquasil Ultra, Dentsply, St. York, PA, USA) impression of the two premolars and the first molar in the mandible were made in accordance with the standard procedure. Crowns with nine different materials with the same size and shape were cast before putting onto the teeth. Titanium implants, abutments were placed after implant oval preparations in the mandibular right premolar region (Fig. 1). Crowns of the implants were fabricated and placed onto the titanium abutments afterwards. The compositions of the nine dental materials used in the dry human mandible were in accordance with those used in the human cadaver.

A plastic container filled with gelatine was used because its signal was similar to that of soft tissue, and a resin holder was applied to help locate the skull or the dry mandible during examination^17,25,26^.

### Imaging

All of the specimens were scanned through MRI (GE Healthcare, Waukesha, WI, USA). The scanning parameter details are listed in Table 2. When analyzing the images, we chose a horizontal plane transverse the zygomatic arch and tubera maxillare as the reference plane in the skull. As for the implant-supported crowns in the mandible, the plane transverse the entry zone of the left and right alveolar nerve tubes were set as the reference plane to ensure the identical planes for consecutive measurements. All of the operations were performed by one professional to ensure that the scanning plane traversed the same coronal position. Each specimen was scanned thrice, and the image files were saved in DICOM for analysis.

**Table 2:** Details of MRI scanning parameters.

Parameter	Sequence	Field of View (FOV)	Thickness (THK)	Spacing (SP)	TR/TE	Matrix	Number of Excitations (NEX)	Echo Train Length (ETL)	Bandwidth (BW)
Details	FRFSE-T2WI	20 mm × 20 mm	3 mm	1.5 mm	2500 ms/106.6 ms	288 × 192 pixels	1	16	25 Hz/pixel

### Image analysis

**Figure 2:**
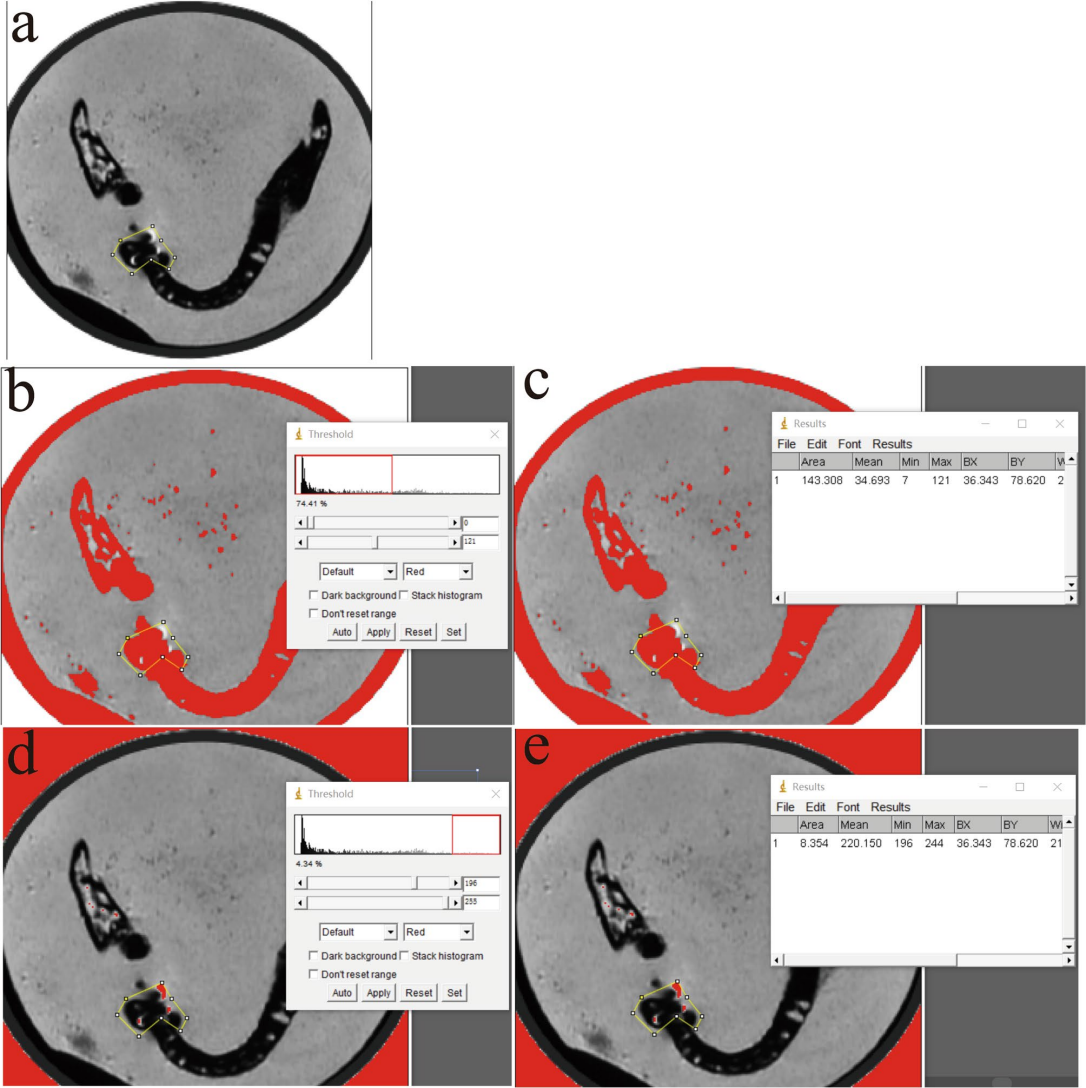
The artifacts areas measurement procedures. The artifacts areas measurement procedures. Selection of the ROI around the implant or the abutment in the image (**a**). Set the threshold (Image-Adjust-Threshold) (**b**, **d**). Measure the artifact areas of signal loss and signal intensity respectively (Analyze-Measurement) (**c**, **e**).

Uncompressed images in DICOM were transferred in ImageJ2x (National Institute of Health, Bethesda, MD, USA). The areas of the artifacts were calculated within the same horizontal plane as the anatomical marks. The region of interest (ROI) was drawn such that it corresponded to a distorted region from visual inspection and was compared with the ROI in the images with no dental materials with the corresponding pixel position^27^. Artifacts were defined as the signal intensity and signal loss adjacent to the prosthesis. For artifact size quantification, the voxels beyond the mean background intensity and three times the respective standard deviation were defined as signal intensity, whereas the voxels within the mean signal loss intensity and three times the respective standard deviation were classified as signal loss according to a previous study^8,21,23,26^. First of all, we selected the ROI around the implant or the abutment in the image (Fig. 2a). Secondly, the threshold was set as the voxels within the mean signal loss intensity and three times the respective standard deviation and beyond the mean background intensity and three times the respective standard deviation (Image-Adjust-Threshold) (Fig. 2b, d). Then the areas were then measured respectively (Analyze-Measurement) (Fig. 2c, e). All of the images were measured by one professional. Each image was examined thrice, and the mean value and deviations were recorded. Twenty images selected randomly were re-measured after 20 days.

### Statistical analysis

Intraclass correlation coefficient (ICC) was employed to evaluate the reliability of the artifact areas of the 20 images measured in 20 days and repetition of the three scans for the same specimen. As resin was reported to induce almost no susceptibility artifacts, the resin crowns were set as control in the following analysis^26^. As for implant group, the blank group was set as control. Meanwhile, one-way ANOVA or t-test analysis was performed to compare multiple means of samples in SPSS 20.0 (Statistical Package for the Social Sciences, v22.0; IBM, Armonk, NY, USA). *P* < 0.05 indicated significant differences.

### Ethical approval and consent to participate

All procedures performed in studies were in accordance with the 1964 Helsinki Declaration and approved by the Medical Ethic Committee of Xiangya Hospital of Central South University (Approval Number: 201610068).

## Results

**Figure 3:**
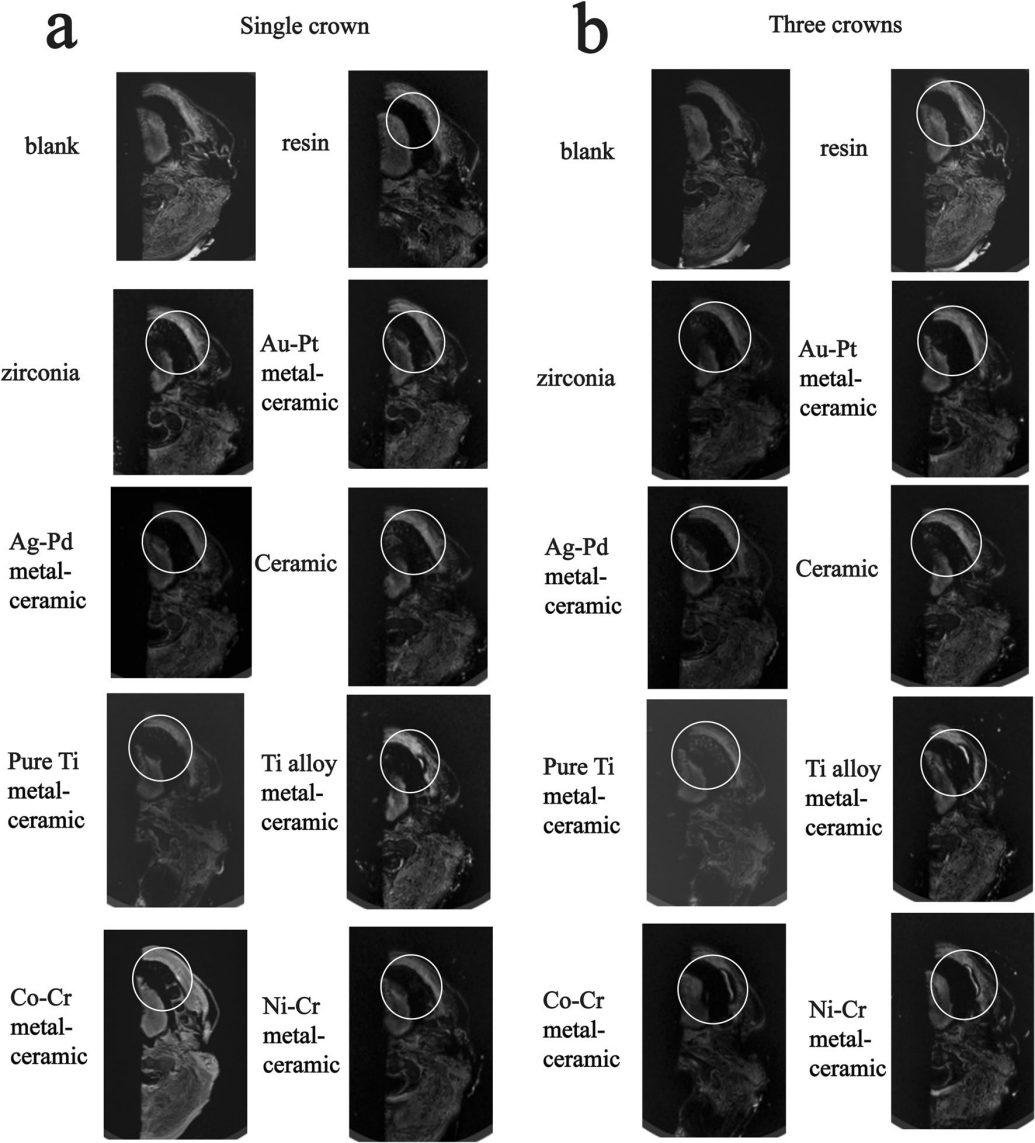
Artifacts induced by single (**a**) or three crowns (**b**) of nine different materials in the human cadaver in FRFSE-T2WI (White circles indicate the areas affected by artifacts).

**Figure 4:**
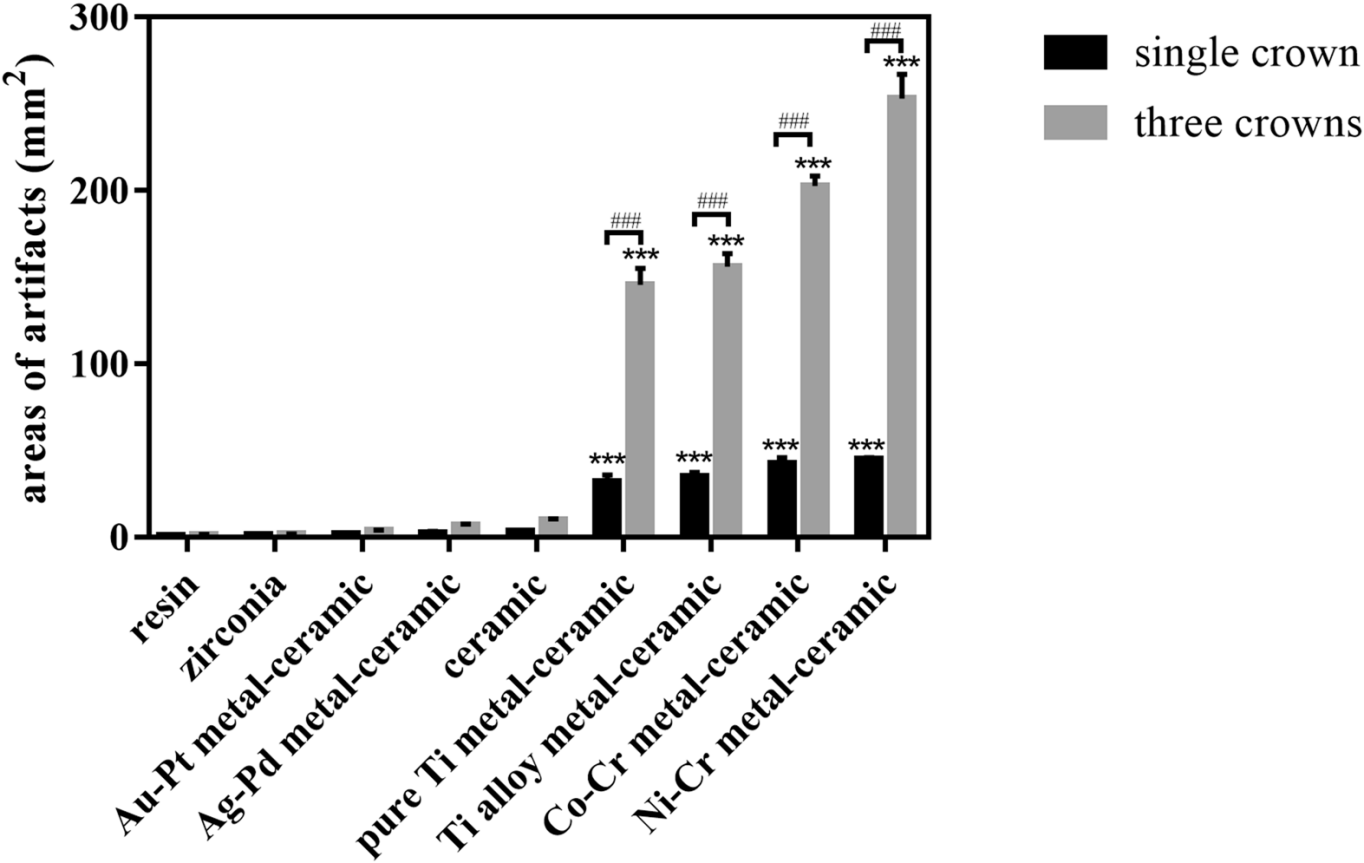
Quantitative analysis of artifacts produced by the single or three crowns of nine different materials. (^#^*P* < 0.05, ^##^*P* ≤ 0.01, and ^###^*P* ≤ 0.001 between each group. *, **, and *** indicate significant differences in comparison with the resin group. * *P* < 0.05, ** *P* ≤ 0.01, and *** *P* ≤ 0.001).

The ICC for multiple images were 0.997 and the ICC for multiple measurements were 0.999. The results revealed that the resin crown barely produced artifacts, and its signal was similar to that of the surrounding soft tissue (Fig. 3a). Precious metal-ceramic (gold-platinum, Au-Pt and silver-palladium, Ag-Pd), ceramic and zirconia crowns produced slight artifacts. Moderate arc-shaped artifacts could be observed around pure Ti (pure titanium) metal-ceramic crowns. The hypodense signal was presented in the lingual sides around the pure Ti metal-ceramic crowns and break the continuity of the lingual images. However, the artifacts did not interfere with the normal structure of peridentium of neighbouring teeth, the buccal tissues or the brain tissues. More artifacts were shown in nonprecious metal-ceramic (titanium alloy, Ti alloy; cobalt-chromium, Co-Cr; nickel–chromium, Ni–Cr) crowns with similar distribution patterns (Fig. 3). Apart from the hypodense signal at the lingual sides, the hyperdense signal was detected at the buccal sides around these crowns, which interfered with the images of buccal soft tissues. Besides, three crowns generated much more image distortion than the single one (Fig. 3b). The quantitative analysis of areas of the artifacts induced by the crown group is presented in Fig. 4. Resin, zirconia, Au-Pt meta-ceramic, Ag-Pd metal-ceramic and ceramic crowns barely produced susceptibility artifacts (p > 0.999). Single pure Ti metal-ceramic, Ti alloy metal ceramic, Co-Cr metal-ceramic, Ni–Cr metal-ceramic crowns produced 31.833 mm^2^—44.616 mm^2^ artifacts around teeth (*p* < 0.001) and when three crowns were placed onto the prepared teeth, the areas of artifacts rose significantly (*p* < 0.001).

**Figure 5:**
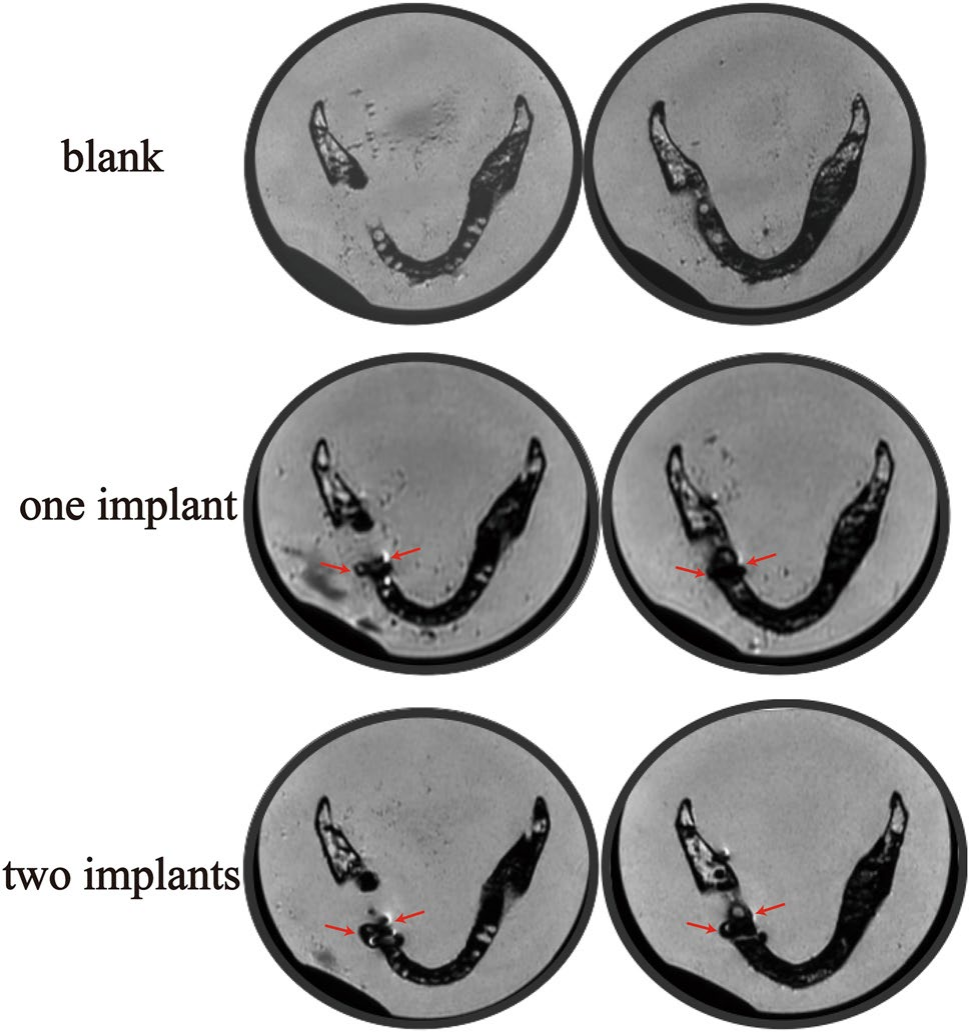
Susceptibility artifacts generated by titanium implants in a dry human mandible FRFSE-T2WI (red arrows indicate the artifacts).

**Figure 6:**
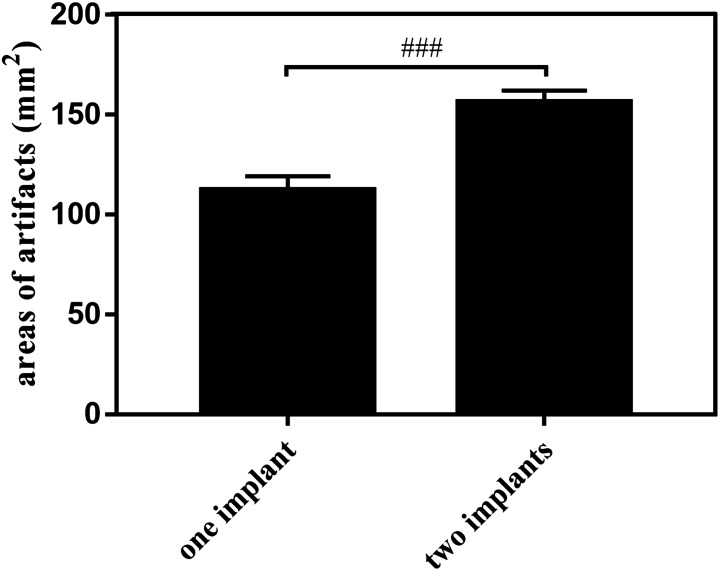
Quantitative analysis of the artifacts induced by titanium implants in the dry human mandible (^###^*P* ≤ 0.001 between each group).

When titanium implants were placed into the mandible, hypodense circular distortion were showed at both the buccal and lingual aspects with hyperdense artifacts detected at the lingual side around the hypodense signal (Fig. 5). The distortion did not appear at the peridentium areas of neighbouring teeth or interfere with the normal structure of other teeth or the mandibular ramus. Quantitative analysis revealed that one implant induced 112.460 ± 6.818 mm^2^ areas of artifacts and 156.407 ± 5.706 mm^2^ areas artifacts were produced after one more implant insertion. The difference was significant (*p* = 0.001) (Fig. 6).

**Figure 7:**
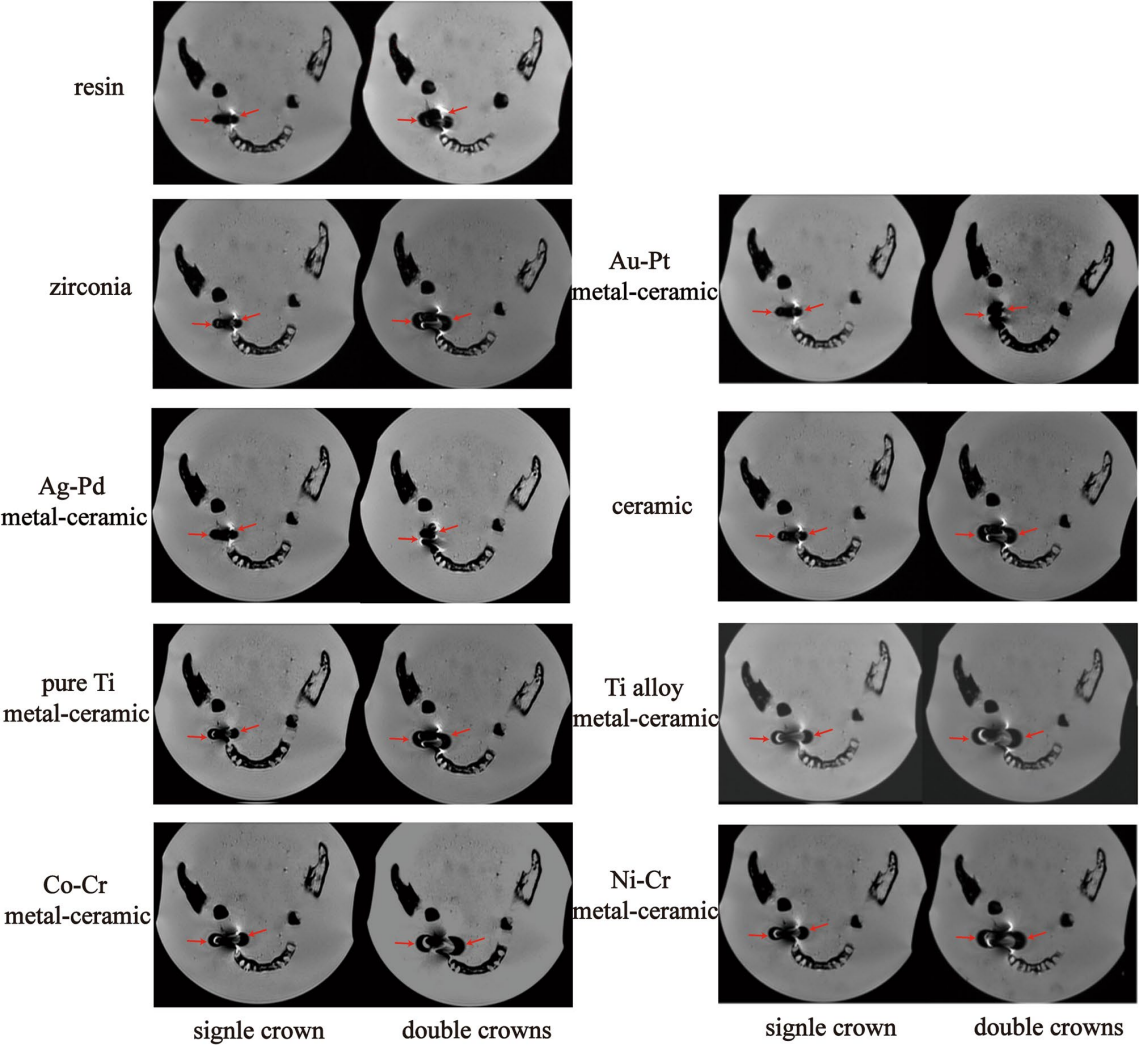
Images of the artifacts produced by one or two titanium restorations with nine different materials in the dry human mandible in FRFSE-T2WI (red arrows indicate the artifacts).

**Figure 8:**
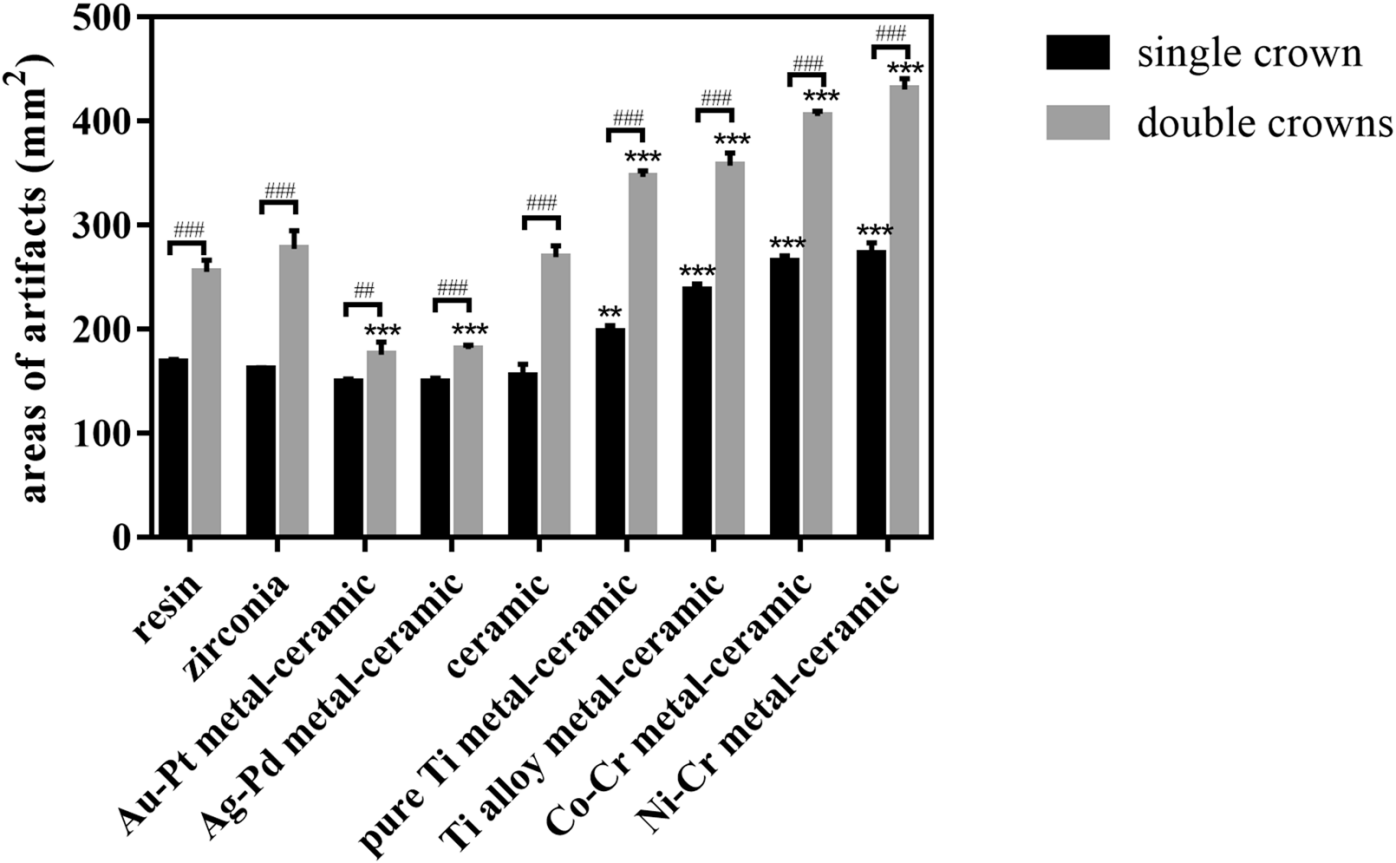
Quantitative analysis of the artifacts produced by the single or double crowns of nine different materials combined with titanium implants and abutments in MRI (^#^*P* < 0.05, ^##^*P* ≤ 0.01, and ^###^*P* ≤ 0.001 between each group. *, **, and *** indicate significant differences in comparison with the resin group. * *P* < 0.05, ** *P* ≤ 0.01, and *** *P* ≤ 0.001).

After the titanium implants and prosthesis abutments were placed, the resin, ceramic and zirconia crown restorations all caused evident image distortion in MRI (Fig. 7). The addition of pure Ti, Ti alloy, Co-Cr and Ni–Cr metal-ceramic crowns increased the areas of artifacts. Artifact areas enlarged as the number of prosthesis increased (Fig. 8). Double Co-Cr and Ni–Cr metal-ceramic crowns increased artifact areas by 150.10 mm^2^ (*p* < 0.001) and 175.50 mm^2^ (*p* < 0.001) respectively. Nevertheless, the least artifacts were observed in the Au-Pt metal-ceramic crowns for the titanium implants. The double Ag-Pd metal-ceramic crowns also presented 79.49 mm^2^ and 74.17 mm^2^ areas artifacts reduction than those induced by the resin crowns and the reduction was significant (*p* < 0.001) when combined with titanium implants (Figs. 7, 8).

## Discussion

Head and neck MRI serves as the most commonly used auxiliary examination for diagnosing tumors and other lesions in the maxillofacial region^1–5^. To investigate the possible artifacts produced by oral prosthesis in MRI, we included commonly-used crowns with nine different materials and titanium implants to compare the susceptibility artifacts. The hypothesis was not rejected in our investigation. Resin, ceramic, zirconia and precious metal-ceramic crowns could produce few artifacts on the prepared teeth. However, when combined with titanium implants, Au-Pt and Ag-Pd metal-ceramic crowns would decrease the artifacts that titanium implants produced. Artifact size was increased in Pure Ti, Ti alloy, Ni–Cr and Co-Cr metal-ceramic crowns.

1.5 T MRI is clinically used for the evaluation of the oral tissue, and this technique was reported to cause fewer susceptibility artifacts than 3 T MRI does at the same imaging parameters for the common dental materials^4,28,29^. Therefore, 1.5 T MRI was applied in the experiment. The susceptibility artifacts of single-metal objects in dentistry have been investigated using in vitro models or a porcine jaw^16,18,22,30^. A previous study has investigated the artifacts induced by four kinds of cylindrically shaped materials. It classified resin, glass ionomer cement as fully compatible materials, amalgam, gold alloy, gold-ceramic materials, titanium causing comparatively strong distortions as compatible materials and steel orthodontic appliances and Co-Cr as non-compatible materials^15^. The resin, zirconia, ceramic and precious metal-ceramic crowns exhibited almost no artifacts according to our investigation. By contrast, the pure Ti metal-ceramic produced moderate artifacts and nonprecious metal-ceramic crowns on the prepared teeth created severely distorted images. In the transverse plane, there was a decrease in signal density at the lingual aspect of the crowns and an increase at the buccal side induced by the Ti alloy porcelain crowns. The distortion patterns were similar in the Co-Cr and Ni–Cr metal-ceramic crowns with larger areas. The artifacts were positively associated with the number of prosthesis. These results were consistent with those of previous studies^15,22,31^.

For the implants placement, we used a dry human mandible with gelatine to create surroundings similar to those of the oral cavity. Given the consistency of gelatine in the phantom, a crown can be located, and the effect of interference with air can be eliminated^31^. Titanium implants could generate moderate artifacts with circular low-dense signal at both the lingual and buccal aspects and high-dense signal at lingual sides. A recent study has also investigated the artifacts induced by titanium implants of different diameters and height. The research found that titanium implants could generate circular or “clover-like” artifacts, which was in consistence with our results^32^.

When full crowns were combined with titanium implants and abutments, different results were observed compared with prepared teeth. Significant artifacts were detected in the crown plane in MRI regardless of the material of the full crowns. The resin crowns induced significant signal voids in the crown plane with the titanium implants and abutments, whereas no artifacts were observed in resin crowns with the prepared teeth. The non-uniformity of the results in the tooth-supported group and the implant-supported group could be attributed to the difference in susceptibility between prepared teeth and titanium abutments. The results suggested that titanium abutments altered the precession frequencies of protons and caused the signal loss and spatial distortions at the adjacent areas.

Nevertheless, the artifacts reduced significantly when titanium implants were combined with precious metal-ceramic crowns. A framework of nonprecious metal-ceramic crowns supported by titanium implants still induced the most evident artifacts. A previous study has observed that precious metal and zirconia crowns are favourable in terms of low artifacts in compared with the nonprecious metal crowns with titanium implants^23^. Furthermore, we have observed the reduction of artifacts in titanium implant-supported Au-Pt and Ag-Pd crowns. When titanium, a paramagnetic material with a susceptibility of 182 ppm, was combined with diamagnetic materials, such as gold (susceptibility: − 34 ppm) and silver (susceptibility: − 24 ppm), whose susceptibilities are stronger than those of resin (susceptibility: − 11 ppm to − 7 ppm), diminished artifacts were noted in the MRI^6,33^. A research also observed reduced artifact size when a paramagnetic NiTi implant was coated with diamagnetic grapheme oxide and carbon nanotubes, which was in consistence with our results^34^. On the contrary, when titanium was united with other paramagnetic materials, such as nickel (susceptibility: 600 ppm), chromium (susceptibility: 320 ppm) and cobalt (susceptibility: 250 ppm), artifacts increased^6,35^. The zirconia and ceramic crowns combined with titanium implants did not show significant artifacts reduction. Even though the susceptibility of silica (susceptibility: − 16.3 ppm) was stronger than resin, ceramic crowns did not reduced artifacts generated by titanium implants significantly. That was probably because ceramic crowns are composed of 70% of silica and 30% of other metal oxides. The addition of other oxides might affect the susceptibility of ceramic crowns, further influenced the results in MRI. The susceptibility of zirconia (susceptibility: − 8.3 ppm) was quite close to that of resin, so no obvious artifacts reduction was observed. Moreover, the difference of the volume and shape between titanium abutments and crowns also influenced the artifacts distribution, which made the situation more complicated^19^. The observation indicated that when an upper restoration was required after titanium implant placement, diamagnetic materials would be favourable for less artifacts in MRI.

In our study, the inconspicuous artifacts were detected in the implant plane but obvious image distortion was shown in the crown plane. That was probably because the disparity in susceptibility between dental materials and surrounding environments were different, as the titanium implant was surrounded by mandibular alveolar bone and the upper restorations were surrounded by gelatine.

This study is characterised by some limitations. A dry human mandible and surrounding gelatine were utilized to compare the artifacts induced by titanium implants and crowns. Gelatine could not represent the anatomic structures in vivo and the susceptibility of gelatine and oral cavity was not identical, which might compromise the results. There was only one set of crowns of different materials. More repetition for the materials should be included in the further research. A small field of view (FOV) was utilized in this study, as a smaller FOV induces less susceptibility artifacts. A larger and more clinically-relevant FOV should be included in the future study. Besides, the scanning parameters may have an effect on the artifact size but only one sequence was utilized in the study. More MRI scanning parameters are needed in the future. Only 1.5 T MRI was investigated in our study and more researches on 3 T MRI should be included to get a more comprehensive result. Besides, the artifact areas were measured by one professional, which might cause some random errors. Further validation in patients investigations with two or more investigators analyzing the images are required in this matter.

In conclusion, resin, zirconia, ceramic, precious metal-ceramic crowns barely produce susceptibility artifacts with prepared teeth and nonprecious metal-ceramic crowns would generate signal loss at the lingual aspect of the crowns and an increase at the buccal side and the artifacts would interfere with the buccal mucosa and tongue. After titanium implants insertion, circular signal voids were generated, whereas Au-Pt and Ag-Pd metal-ceramic crowns could alleviate the susceptibility artifacts.

## Abbreviations

MRI: Magnetic resonance imaging
ICC: Intraclass correlation coefficient
Au-Pt: Gold-platinum
Ag-Pd: Silver-palladium
Ti: Titanium
Co-Cr: Cobalt-chromium
Ni–Cr: Nickel–chromium
